# Renal function impairment in children with intestinal failure receiving parenteral nutrition: A descriptive cohort study

**DOI:** 10.1002/jpen.70059

**Published:** 2026-01-25

**Authors:** Amanda M. Braga da Mata, Heitor Pons Leite, Eduardo F. Hatanaka, Mariana Janiques Barcia Magalhães Fonseca, Marta Liliane de Almeida Maia, Giovana S. G. Sabio, Maria Fernanda C. de Camargo, Paulo C. Koch Nogueira

**Affiliations:** ^1^ Department of Pediatrics Universidade Federal de São Paulo São Paulo Brazil; ^2^ Center for Intestinal Rehabilitation Hospital Samaritano de São São Paulo Brazil

**Keywords:** child, intestinal failure, kidney diseases, kidney tubules/abnormalities, pediatrics

## Abstract

**Background:**

Children with intestinal failure are at risk for kidney dysfunction; however, the contributing factors are not well established. We aimed to describe risk factors associated with glomerular and tubular renal dysfunction in children with intestinal failure.

**Methods:**

This retrospective cohort study assessed glomerular and tubular dysfunction in children receiving parenteral nutrition for intestinal failure at two subsequent time points. Glomerular function was assessed using the estimated glomerular filtration rate and urinary microalbumin/creatinine ratio. Tubular function was assessed using the urinary calcium/creatinine ratio and maximum tubular reabsorption of phosphate for estimated glomerular filtration rate. We tested the association between outcomes and ultrashort bowel, age at admission to the intestinal rehabilitation program, length of follow‐up, prematurity, low birth weight, malnutrition, preserved colon in continuity, and ileocecal valve.

**Results:**

The study included 25 patients with a median age of 0.3 (0–14) years, of whom 14 (56%) were boys. Two patients had decreased estimated glomerular filtration rate (one at each time point). Microalbuminuria was observed in four children at time point 1 and persisted in one child at time point 2. Hypercalciuria occurred in 10 of 23 and 9 of 24 children at time points 1 and 2, respectively; 5 maintained this alteration at both time points. Reduced maximum tubular phosphate reabsorption/estimated glomerular filtration rate ratios were observed in 6 of 12 and 2 of 14 children at time points 1 and 2, respectively.

**Conclusion:**

Two‐thirds of children with intestinal failure had renal dysfunction, represented by microalbuminuria, reduced estimated glomerular filtration rate, hypercalciuria, and phosphaturia.

## INTRODUCTION

Children with intestinal failure are at risk of renal impairment. Previous research has identified alterations in glomerular function, as evidenced by a reduced glomerular filtration rate^1^ and proteinuria,^2^ as well as alterations in tubular function, indicated by hypercalciuria^1^ in children with intestinal failure. Overall, the studies conducted thus far highlight the likely multifactorial origins of renal dysfunction in children with intestinal failure.^1^, ^2^ Several predisposing conditions have been identified as potential risk factors: dehydration and hypovolemia, which result from chronic diarrhea—the primary clinical manifestation of extensive intestinal resection—along with the use of nephrotoxic drugs, may threaten renal function in children with intestinal failure.^3^, ^4^

In addition to the aforementioned factors, prematurity and low birth weight, which are prevalent in children who develop short bowel syndrome during the neonatal period, can lead to a reduced number and lower maturation of nephrons at birth.^5^, ^6^, ^7^ Malnutrition resulting from nutrient absorption deficiency is another condition that can affect kidney function in children with intestinal failure. Dysfunctions documented in the literature among severely malnourished children include a decreased glomerular filtration rate, reduced urine concentration capacity, and metabolic acidosis.^8^

Although underlying conditions have been individually identified as risk factors for impaired renal function, there is very limited knowledge on renal function in children with intestinal failure who are monitored over time, particularly concerning the potential effects of prematurity, low birth weight, and malnutrition. In this cohort study, we aimed to describe risk factors associated with glomerular and tubular renal dysfunction in children with intestinal failure.

## METHODS

We performed a descriptive cohort study in 25 children with intestinal failure who received parenteral nutrition at the Intestinal Rehabilitation Centre of Samaritano Hospital in São Paulo from 2019 to 2023. Because of the low prevalence of intestinal failure, a convenience sampling method was used. Children with intestinal failure, with a median age at admission of 0.3 years (range, 0–14 years), who had received home parenteral nutrition for at least 6 months before renal function assessment were included. One patient with underlying nephropathy at the start of follow‐up was excluded. Our study was approved by the Research Ethics Committee of the Samaritan Hospital (protocol 48766621.0.0000.5487), adhering to the Declaration of Helsinki principles. Written informed consent was obtained from patients and/or their authorized representatives.

Patient demographics (sex, gestational age at birth, and current age) and clinical, anthropometric, and laboratory data were extracted from a prospectively collected database of patients in the intestinal rehabilitation program. Patients were characterized by age at diagnosis, etiology of intestinal failure, anatomical classification of short bowel syndrome^9^ (when applicable), length of remaining bowel, presence of colon and ileocecal valve, and follow‐up duration in the program.

The patients’ weight and height were assessed on admission to the program and serially at two subsequent times. Birth weight was recorded and categorized as low weight (yes/no) according to the World Health Organization (WHO).^10^ For nutrition status classification, weight and height‐for‐age *Z* scores were calculated according to the reference values for growth indicators proposed by the WHO^11^, ^12^ using Antro software (for children aged 0 to 5 years) and Antro Plus software (for children >5 years) version 3.2.2. Malnutrition and severe malnutrition were defined by weight and/or height‐for‐age *Z* scores ≤−2.0 and ≤−3.0, respectively.

A multidisciplinary team provided support to patients during both hospitalization and home care. Nutrition support was administered through both parenteral and enteral methods (via tube or orally), and energy, protein, and micronutrient requirements were calculated based on the Dietary Reference Intakes.^13^, ^14^ All patients received home parenteral nutrition containing solutions of vitamins, trace elements, and electrolytes as recommended by the current guidelines of the American Society for Parenteral and Enteral Nutrition (ASPEN).^15^, ^16^

Renal dysfunction (tubular and glomerular) was assessed at two subsequent time points, at least 6 months apart, using paired blood and urine samples. The time interval between the laboratory analyses varied according to the availability of the parameters in the program database. To assess glomerular function, the estimated glomerular filtration rate was calculated based on serum creatinine and height, according to the Schwartz formula,^17^ and microalbumin and creatinine concentrations were measured in a morning urine sample. Tubular function was assessed using simultaneous concentrations of biochemical parameters in the blood and urine. Potential associations with the eight risk factors were tested for both outcomes.

Two coprimary outcomes were specified. The first coprimary outcome glomerular dysfunction was a composite of estimated glomerular filtration rate <90 ml/min/1.73 m²^18^ or microalbuminuria, defined by urinary microalbumin/creatinine ratio >30 mg/g.^19^ Our secondary outcome was tubular dysfunction defined as a composite of hypercalciuria defined by a urinary calcium/creatinine ratio higher than the reference value by age group (0–1 year, <0.81; 1–2 years, <0.56; 2–3 years, <0.50; 3–5 years, <0.41; 5–7 years, <0.30; 7–10 years, <0.25; 10–17 years, <0.24)^20^ or low maximum tubular reabsorption of phosphate for the estimated glomerular filtration rate with values by age group (0–5 months, 3.16–6.19 mg/dl; 6–12 months, 3.50–5.82 mg/dl; 1–5 years, 3.25–5.51 mg/dl, 6–12 years, 3.00–5.08 mg/dl; 13–15 years, 2.82–5.20 mg/dl; >16 years, 2.60–3.80 mg/dl).^21^, ^22^, ^23^, ^24^

The potential risk factors for both outcomes were as follows:

- Ultrashort bowel, defined as a residual bowel length <10 cm^25^;
- Age at admission to the intestinal rehabilitation program;
- Follow‐up period in the program, defined as the time in years from admission to the multidisciplinary intestinal rehabilitation program until the last assessment of the study;
- Prematurity, defined as a positive binary variable if gestational age at birth <37 weeks^7^;
- Low birth weight, defined as a positive binary variable if birth weight <2.5 kg;
- Malnutrition, defined as a positive binary variable if weight or height‐for‐age *Z* scores were ≤−2.11^11^, ^12^;
- Preserved colon in continuity, defined as a positive binary variable if the remaining colon was totally or partially preserved;
- Preserved ileocecal valve, defined as a binary variable.

Qualitative variables were expressed as absolute (*n*) and relative (%) frequencies, and quantitative variables are summarized as medians and interquartile ranges (IQRs). To test the association of risk factors with outcomes, the Mann‐Whitney *U* test was used to compare numerical variables between groups, whereas Fisher's exact test was used for qualitative variables. We specified alpha allocation to adjust the type I error rate for two coprimary outcomes. To maintain an overall type I error rate of 5% (two‐sided), an alpha of 2.5% was allocated to test the coprimary outcome glomerular dysfunction, and 2.5% was allocated to test the coprimary outcome tubular dysfunction. All statistical procedures were carried out using the R language (version 4.2.1) and the R Studio (version 2023.06.2+561).

## RESULTS

The demographic and clinical characteristics of the 25 patients who met the inclusion criteria are shown in Table 1. The median time between admission to the program and the first renal function assessment was 27 (range, 7–44) months, and the median time between the first and second kidney function test was 8 months (range, 6–11 months). The median age at the first and second assessments was 4.5 (IQR, 1.9–5.8) and 5.5 (IQR, 2.9–6.5) years, respectively. At the time of the first and second assessments, only three and two children, respectively, were <1 year of age.

**Table 1:** Clinical and demographic characteristics of patients.

Variables	Values
Follow‐up period, median (IQR), years	4.2 (3.0–5.2)
Age at admission, median (IQR), years	0.3 (0–14)
Male sex, *n* (%)	14 (56)
Preterm, *n* (%)	14 (56)
Gestational age, median (IQR), weeks	6 (32–38)
Birth weight, median (IQR), kg	2.4 (1.4–2.8)
Low birth weight, *n* (%)	15 (60)
Cause of intestinal failure, *n* (%)	
Short bowel syndrome	20 (80)
Hirschsprung's disease	2 (8)
Microvillus inclusion disease	1 (4)
Other	2 (8)
Cause of short bowel syndrome, *n* (%)	
Necrotizing enterocolitis	10 (40)
Volvulus	4 (16)
Gastroschisis	2 (8)
Other	9 (36)
SBS type (*n* = 22; data unavailable in 3), *n* (%)	
Enterostomy	1 (5)
Jejuno‐colic	17 (77)
Jejuno‐ileo‐colic	4 (18)
Ultrashort bowel, *n* (%)	5 (20)
Preserved ileocecal valve (*n* = 24; n/a = 1), *n* (%)	6 (25)
Preserved colon in continuity (*n* = 25), *n* (%)	18 (72)

Abbreviations: IQR, interquartile range; n/a, not applicable; SBS, short bowel syndrome.

On admission, most patients were underweight (*n* = 16; 64%) and short for their age (*n* = 19; 76%). At time point 1 of the renal function assessment, of the 24 (96%) patients with available anthropometric data, 15 (63%) were malnourished, and at time point 2, 13 patients were malnourished (54%). Severe malnutrition was identified in eight (33%) and six (25%) patients at time points 1 and 2, respectively.

During the follow‐up period, the mean energy, protein, and lipid intake supplied by parenteral nutrition for the entire group of children followed in the service were 60.8 (SD, 18.1) kcal/kg/day, 1.8 (SD, 0.5) g/kg/day, and 1.7 (SD, 0.5) g/kg/day, respectively. Patients received calcium and phosphorus as part of parenteral nutrition in the following amounts: calcium was administered at median 0.59 (IQR, 0.33–0.54) mEq/kg/day at the first time point and median 0.66 (IQR, 0.51–0.70) mEq/kg/day at the second time point, whereas phosphorus was given at median 0.63 (IQR, 0.23–1.07) mmol/kg/day at the first time point and median 0.63 (IQR, 0.25–1.27) mmol/kg/day at the second time point.

### Changes in kidney function

Considering only evaluable cases, glomerular dysfunction was identified in 5 of 22 children (22.7%) at the first and in 1 of 24 (4.2%) at the second assessment, whereas tubular dysfunction was present in 13 of 23 (56.5%) and 9 of 24 (37.5%), respectively. Three children exhibited both glomerular and tubular abnormalities at the first assessment and none at the second. Persistent abnormalities were observed in one child for glomerular dysfunction and in five for tubular dysfunction, as represented in Table 2.

**Table 2:** Parameters of glomerular and tubular function in the two time points of the study.

Glomerular function	Time point 1	Time point 2
eGFR, median (IQR), ml/min/1.73 m²	129 (110–148) [*n* = 21]	129 (105–151) [*n* = 24]
Decreased eGFR, median (IQR)	1 (4.8) [*n* = 21]	1 (4.2) [*n* = 24]
Microalbumin/creatinine, median (IQR), mg/g	16 (7.8–29.2) [*n* = 20]	21 (8–29) [*n* = 15]
Increased microalbuminuria, *n* (%)	4 (20.0) [*n* = 20]	1 (6.7) [*n* = 15]
Glomerular dysfunction, *n* (%)	5 (22.7) [*n* = 22]	1 (4.2) [*n* = 24]
Tubular function		
Urinary Ca/Cr, median (IQR), mg/mg	0.3 (0.1–0.1) [*n* = 23]	0.3 (0.2–0.6) [*n* = 24]
Hypercalciuria, *n* (%)	10 (43.5) [*n* = 23]	9 (37.5) [*n* = 24]
TmP/GFR, median (IQR), mg/dl	3.3 (2.6–4.3) [*n* = 12]	3.9 (3.4–4.3) [*n* = 14]
Decreased PO₄ reabsorption, *n* (%)	6 (50.0) [*n* = 12]	2 (14.3) [*n* = 14]
Tubular dysfunction, *n* (%)	13 (56.5) [*n* = 23]	9 (37.5) [*n* = 24]

*Note*: Percentages were calculated over the number of patients with available data (*n*) at each time point. For glomerular and tubular dysfunction, the denominator corresponds to the total number of patients with at least one of the component tests available.

Abbreviations: Ca, Calcium; Cr, Creatinine; eGFR, estimated glomerular filtration rate; IQR, interquartile range; N/A, data not available; PO_4_, phosphate; TmP/GFR, maximum tubular phosphate reabsorption for glomerular filtration rate.

When data from both assessments were combined, 17 of 25 children (68%) had at least one renal abnormality—glomerular, tubular, or both—during follow‐up.

Among the risk factors studied, only ultrashort bowel had a significant association with the outcome glomerular dysfunction at time point 1 because 12 out of 13 (92%) cases with this outcome had an ultrashort bowel, whereas only 4 out of 10 (40%) without glomerular dysfunction had the same remnant bowel length (Fisher's exact test; *P* = 0.02). At both time points, tubular dysfunction was not significantly associated with any of the listed risk factors.

To better characterize children with tubular dysfunction, we compared serum concentrations of calcium, phosphorus, parathyroid hormone, alkaline phosphatase, blood pH, sodium, and chlorine between patients who had dysfunction and those who never had tubular dysfunction. The results are presented in Tables S1 and S2.

## DISCUSSION

At least one alteration in kidney function was detected in 68% of children with intestinal failure at the time points of the study. Considering glomerular function alone, five patients (25%) had altered tests at the first time point (one patient with low estimated glomerular filtration rate and four children with microalbuminuria) and two at the second time point (one patient with low estimated glomerular filtration rate and one with microalbuminuria). In cases of glomerular dysfunction, a slight drop in estimated glomerular filtration rate was observed in a few cases, and microalbuminuria was observed in a slightly higher proportion of cases. Therefore, at some time point during the follow‐up, a quarter of the children showed a pattern of mild glomerular involvement, characterized by excess microalbumin in the urine or a decrease in estimated glomerular filtration rate. However, these changes were not long‐lasting, except in one case, and may represent transient fluctuations in glomerular function over time. Our findings are similar to those of Messova et al^26^ who followed 25 children with intestinal failure for 3 years and found no significant decline in estimated glomerular filtration rate during this period, even though some cases (8%–25% of the sample) showed drops in this parameter at each of the five time points of their study. These fluctuations may have enduring implications for renal function, which can only be confirmed through more extended longitudinal studies.

When examining estimated glomerular filtration rate values, the occurrence of cases suggestive of glomerular hyperfiltration (filtration rate >140 ml/min/1.73 m^2^) is noteworthy, with 8 children affected at time point 1 and 11 children at time point 2. Determining the clinical significance of this finding is challenging because malnutrition prevalence among patients was nearly half the sample at both time points (44% and 40%, respectively); consequently, elevated estimated glomerular filtration rate values may result from overestimating glomerular filtration rate based on creatinine dosage. Malnutrition is associated with loss of muscle tissue, which is the main producer of creatinine. With muscle mass depletion, creatinine production decreases, which can lead to the false impression of increased glomerular filtration.^27^, ^28^, ^29^ It is worth noting that 21 of 25 patients in the sample were admitted with malnutrition, and 11 of 25 and 10 of 25 children were still malnourished at time points 1 and 2, respectively. In the presence of malnutrition, cystatin levels, together with microalbuminuria, are more appropriate markers of glomerular function.^30^, ^31^ In the present study, we did not report cystatin C measurements because this test was not part of the routine laboratory assessment at our center during the data collection period.

In addition to the assessment of estimated glomerular filtration rate, the presence of microalbuminuria in four and one cases at the respective time points of the study indicates that certain patients showed some degree of glomerular hypertension. This alteration is significant because it may serve as a biomarker for kidney disease, with the potential to progress into more severe disorders over time.^32^ A similar phenomenon was detected by Billing et al^2^ and later by Mark et al,^33^ who showed glomerular proteinuria. In the study by Mark et al,^33^ urinary protein loss correlated positively with the parenteral supply of amino acids; however, as in our study, there was no association between glomerular dysfunction and the duration of parenteral nutrition.

Alterations in tubular function, detected in 13 children at the first and 10 children at the second time point, were mild and characterized mainly by hypercalciuria and a low rate of tubular reabsorption of phosphate, which was only assessed in 56% of the sample. The increased urinary excretion of calcium and phosphorus warrants attention, and interpreting these findings is complex, given that phosphorus reabsorption is confined to the proximal tubule, whereas calcium reabsorption occurs in the proximal tubule, the loop of Henle, and the distal tubule. It is unlikely that a specific tubulopathy is responsible for the two isolated findings, suggesting that they may be the result of functional tubular variations, probably secondary to the supply of electrolytes, osmolarity of the infused solutions, use of medication, or even transient metabolic and energetic disturbances in renal tubular cells associated with intestinal failure.^34^

In relation to hypercalciuria, recurrent dehydration episodes resulting from diarrhea, along with electrolyte and acid‐base imbalances, are linked to nephrocalcinosis in intestinal failure. The hypercalciuria identified in the patients of our study led to a review of previous ultrasound images, which revealed no instances of nephrocalcinosis. This observation aligns with the findings of Mark et al,^33^ who, despite noting a high prevalence (50%) of hypercalciuria among the children with intestinal failure assessed (*n* = 14), identified nephrocalcinosis in only one case. Billing et al^2^ studied 50 patients with intestinal failure and found that hypercalciuria, which was present in 60% of the sample, was associated with excessive parenteral calcium supplementation. In the analysis of the prescriptions for patients in our study (data not shown) at the two time points when renal function was assessed, excessive calcium supplementation via parenteral nutrition was not detected, and the calcium daily supply was in line with the recommendations of the European Society of Pediatric Gastroenterology, Hepatology and Nutrition (ESPGHAN).^35^ This allowed us to infer that the hypercalciuria identified in this study is suggestive of reduced tubular calcium reabsorption.

Among the studies assessing renal function in children with intestinal failure, only the investigation conducted by Billing et al^2^ examined urinary phosphorus loss. However, they did not report the proportion of patients who experienced this alteration; it was only highlighted that this finding was more prevalent in children with chronic kidney disease. Although we observed hyperphosphaturia in some patients, chronic kidney disease did not occur and the daily supply of phosphorus via parenteral nutrition (data not shown) was not excessive, in accordance with the recommendations of the ESPGHAN.^35^

The higher frequency of renal dysfunction (68%) observed in our study compared with previously published studies^1^, ^2^, ^33^ can probably be attributed to the younger age and less favorable short bowel syndrome type of the patients in our sample, which included a greater number of patients with ultrashort bowel (20%) and fewer with a preserved ileocecal valve (25%).

The main limitations of this study were its retrospective design, which prevented the availability of data from all participants; its observational nature, which precludes the establishment of causal relationships; the small sample size, which may have been insufficient to detect certain associations; and the fact that the potential association between parenteral nutrition composition and renal function outcomes was not tested. Nevertheless, we assessed renal function at two separate time points during the follow‐up period. The longitudinal analysis revealed that certain changes in renal function observed in these children may be transient, thereby contributing to a deeper understanding of renal function in children with intestinal failure.

## CONCLUSION

Approximately two‐thirds of children with intestinal failure develop glomerular and tubular dysfunction, as evidenced in this study by microalbuminuria, reduced estimated glomerular filtration rate, and urinary loss of calcium and phosphorus, respectively. These results show that children with intestinal failure are at risk for renal function impairment, which may be influenced by the severity of the primary intestinal disease. Consequently, routine clinical management of these patients should incorporate assessment of both glomerular and tubular functions. Future research should focus on long‐term evaluation of renal function and assessment of bone mineralization in this patient population.

## AUTHOR CONTRIBUTIONS

Amanda M. Braga da Mata, Paulo C. Koch Nogueira, and Heitor Pons Leite conceived the study. Amanda M. Braga da Mata, Eduardo F. Hatanaka, Mariana Janiques Barcia Magalhães Fonseca, and Giovana S. G. Sabio collected the data. Amanda M. Braga da Mata, Paulo C. Koch Nogueira, Heitor Pons Leite, Eduardo F. Hatanaka, and Marta Liliane de Almeida Maia analyzed the data and interpreted the results. Amanda M. Braga da Mata, Paulo C. Koch Nogueira, and Heitor Pons Leite drafted and revised the study. All authors discussed the results and contributed to the final manuscript.

## CONFLICT OF INTEREST STATEMENT

None declared.

## Supporting information

Supplementary tables.
