# Supplementary material for: Renal function impairment in children with intestinal failure receiving parenteral nutrition: A descriptive cohort study
Source: JPEN J Parenter Enteral Nutr. 2026 Jan 25;50(4):515–20. doi: 10.1002/jpen.70059 (PMC13169219; doi:10.1002/jpen.70059)
Supplement: Supplementary file 1 — Supplementary tables. [file JPEN-50-515-s001.dotx]

**Supplementary tables**

**Supplementary Table 1** – Laboratory parameters according to urine calcium concentration at the two time points of the study

**Time point 1 Time point 2**

| Variable | | Urinary calcium (n) | | Median (IQR) | p | | Urinary calcium (n) | Median (IQR) | p | |
| --- | --- | --- | --- | --- | --- | --- | --- | --- | --- | --- |
| Serum calcium | | High (9) | | 1.2 (1.2-1.3) |  | | High (9) | 1.3 (1.3-1.3) |  | |
|  | | Normal (15) | | 1.3 (1.2-1.3) | 0.419 | | High (14) | 1.3 (1.3-1.3) | 0.155 | |
| Serum phosphorus | | High (9) | | 3.8 (4.3-5.0) |  | | High (9) | 4.2 (3.9-4.5) |  | |
|  | | Normal (15) | | 4.6 (3.7-4.6) | 0.385 | | Normal (15) | 4.7 (4.0-5.1) | 0.160 | |
| Serum magnesium | | High (9) | | 1.8 (1.7-1.9) |  | | High (8) | 1.7 (1.7-1.8) |  | |
|  | | High (15) | | 1.9 (1.8-2.1) | 0.062 | | Normal (15) | 1.8 (1.8-2.0) | **0.034*** | |
| Serum Chloride | | High (7) | | 102 (101-103) |  | | High 7) | 103 (102-104) |  | |
|  | | High (14) | | 103 (102-105) | 0.281 | | Normal (14) | 103 (102-105) | 0.567 | |
| PTH | | High (8) | | 39 (36-42) |  | | High (7) | 48 (46-48) |  | |
|  | | Normal (6) | | 32 (23-32) | 0.222 | | Normal (7) | 37 (36-40) | 0.165 | |
| Alkaline phosphatase | | High (9) | | 342 (212-338) |  | | High (9) | 289 (179-308) |  | |
|  | | Normal (14) | | 265 (227-437) | 0.456 | | Normal (14) | 230 (169-272) | 0.705 | |
| Bicarbonate | | High (8) | | 25 (24-25) |  | | High (8) | 24 (24-24) |  | |
|  | | Normal (15) | | 24 (23-25) | 0.807 | | Normal (15) | 22 (22-24) | 0.060 | |
| Blood pH | | High (8) | | 7.39 (7.39-7.40) |  | | High (8) | 7.37 (7.37-7.40) |  | |
|  | | Normal (15) | | 7.39 (7,37-7.39) | 0.187 | | High (15) | 7.37 (7.36-7.38) | 0.433 | |
| Urine density | | High (8) | | 1.010 (1.010-1.010) |  | | High (7) | 1.020 (1.010-1.020) |  | |
|  | | Normal (13) | 1.010 (1.010-1.010) | | 0.785 | Normal (13) | 1.010 (1.000-1.010) | | 0.046 | |

* Change that is both statistically and clinically significant **(?)**

**Supplementary Table 2** –Laboratory parameters according to maximum tubular phosphate reabsorption for glomerular filtration rate (TmP/GFR) at both study time points

**Time point 1 Time point 2**

| Variable | TmP/GFR (n) | Median (IQR) | p value | TmP/GFR (n) | Median (IQR) | p value |
| --- | --- | --- | --- | --- | --- | --- |
| Serum calcium | Low (6) | 1.2 (1.2-1.2) |  | Low (2) | 1.4 (N/A-N/A) |  |
|  | Normal (6) | 1.3 (1.2-1.3) | 0.108 | Normal (12) | 1.3 (1.3-1.3) | 0.100 |
| Serum phosphorus | Low (6) | 3.5 (3.3-3.8) |  | Low (2) | 2.5 (N/A-N/A) |  |
|  | Normal (6) | 4.6 (4.2-4.7) | **0.024*** | Normal (12) | 4.5 (4.3-4.7) | **0.035*** |
| Serum magnesium | Low (6) | 1.8 (1.8-1.8) |  | Low (2) | 1.7 (N/A-N/A) |  |
|  | Normal (6) | 1.9 (1.8-2.1) | 0.405 | Normal (12) | 1.8 (1.8-2.1) | 0.107 |
| Serum Chloride | Low (6) | 103 (102-103) |  | Low (2) | 104 (N/A-N/A) |  |
|  | Normal (5) | 104 (102-105) | 0.511 | Normal (11) | 103 (102-104) | 0.366 |
| PTH | Low (6) | 30 (20-41) |  | Low (0) | N/A |  |
|  | Normal (3) | 41 (32-87) | 0.575 | Normal (6) | 40 (35-39) | N/A |
| Alkaline phosphatase | Low (6) | 266 (174-353) |  | Low (1) | 810 (N/A-N/A) |  |
|  | Normal (5) | 240 (229-246) | 1.000 | Normal (12) | 261 (188-318) | 0.141 |
| Bicarbonate | Low (5) | 24 (24-25) |  | Low (2) | 23 (N/A-N/A) |  |
|  | Normal (6) | 24 (24-25) | 1.000 | Normal (11) | 23 (23-25) | 0.541 |
| Blood pH | Low (5) | 7.39 (7.38-7.41) |  | Low (2) | 7.39 (N/A-N/A) |  |
|  | Normal (6) | 7.38 (7.37-7.39) | 0.139 | Normal (11) | 7.37 (7.36-7.37) | 0.189 |
| Urine density | Low (6) | 1.010 (1.010-1.010) |  | Low (2) | 1.010 (N/A-N/A) |  |
|  | Normal (5) | 1.010 (1.010-1.020) | 0.302 | Normal (12) | 1.010 (1.010-1.020) | 1 |

N/A – Not applicable; * change that is both statistically and clinically significant
